# Diagnostic Accuracy of Abdominal CT for Locally Advanced Colon Tumors: Can We Really Entrust Certain Decisions to the Reliability of CT?

**DOI:** 10.3390/jcm12216764

**Published:** 2023-10-26

**Authors:** Yaiza García del Álamo Hernández, Óscar Cano-Valderrama, Carlos Cerdán-Santacruz, Fernando Pereira Pérez, Inés Aldrey Cao, Sandra Núñez Fernández, Eduardo Álvarez Sarrado, Rosángela Obregón Reina, Paula Dujovne Lindenbaum, María Taboada Ameneiro, David Ambrona Zafra, Silvia Pérez Farré, Marta Pascual Damieta, Ricardo Frago Montanuy, Blas Flor Lorente, Sebastiano Biondo, Collaborative Group for the Study of Metachronous Peritoneal Metastases of pT4 Colon Cancer

**Affiliations:** 1 Colorectal Surgery Department, Hospital Universitario de la Princesa, Instituto de Investigación Sanitaria Princesa (IIS-IP), Universidad Autónoma de Madrid (UAM), 28006 Madrid, Spain; 2 Colorectal Surgery Department, Complejo Hospitalario Universitario de Vigo, 36312 Vigo, Spain; oscarcanovalderrama@hotmail.com; 3 General Surgery Department, Hospital de Fuenlabrada, 28942 Madrid, Spain; fernando.pereira@salud.madrid.org; 4 Colorectal Surgery Department, Complexo Hospitalario Universitario de Ourense, 32005 Ourense, Spain; inesaldrey@hotmail.com (I.A.C.);; 5 Colorectal Surgery Department, Hospital Politécnico Universitario la Fe, 46026 Valencia, Spain; 6 Colorectal Surgery Department, Hospital General Universitario Gregorio Marañón, 28007 Madrid, Spain; 7 Colorectal Surgery Department, Complejo Hospitalario Universitario de A Coruña (CHUAC), 15006 A Coruña, Spain; maria.taboada.ameneiro@sergas.es; 8 Colorectal Surgery Department, Hospital Arnau de Vilanova de Lleida, 25198 Lleida, Spain; 9 Colorectal Surgery Department, Hospital del Mar de Barcelona, 08003 Barcelona, Spain; mpascual@psmar.cat; 10 Department of General and Digestive Surgery, Bellvitge University Hospital, University of Barcelona and IDIBELL, 08908 L’Hospitalet de Llobregat, Spainsbn.biondo@gmail.com (S.B.); 11 Spanish Surgical Society Colorectal and Peritoneal Surgery Sections, 28006 Madrid, Spain

**Keywords:** advanced colon cancer, CT staging, diagnostic accuracy

## Abstract

Many different options of neoadjuvant treatments for advanced colon cancer are emerging. An accurate preoperative staging is crucial to select the most appropriate treatment option. A retrospective study was carried out on a national series of operated patients with T4 tumors. Considering the anatomo-pathological analysis of the surgical specimen as the gold standard, a diagnostic accuracy study was carried out on the variables T and N staging and the presence of peritoneal metastases (M1c). The parameters calculated were sensitivity, specificity, positive and negative predictive values, and positive and negative likelihood ratios, as well as the overall accuracy. A total of 50 centers participated in the study in which 1950 patients were analyzed. The sensitivity of CT for correct staging of T4 colon tumors was 57%. Regarding N staging, the overall accuracy was 63%, with a sensitivity of 64% and a specificity of 62%; however, the positive and negative likelihood ratios were 1.7 and 0.58, respectively. For the diagnosis of peritoneal metastases, the accuracy was 94.8%, with a sensitivity of 40% and specificity of 98%; in the case of peritoneal metastases, the positive and negative likelihood ratios were 24.4 and 0.61, respectively. The diagnostic accuracy of CT in the setting of advanced colon cancer still has some shortcomings for accurate diagnosis of stage T4, correct classification of lymph nodes, and preoperative detection of peritoneal metastases.

## 1. Introduction

Up to 20–30% of colon cancer is diagnosed at locally advanced stages. Although the definition of this group of tumors is not unanimous, it generally comprises tumors classified as T3 with invasion of muscularis propria ≥ 5 mm, T4 with serosal involvement or direct invasion of adjacent structures, and tumors with regional nodal extension [1]. Certain patterns of peritoneal involvement have also been considered in this group of tumors due to similarities in prognosis [2]. Although it is obvious, it has to be taken into account that patients with peritoneal metastases is quite a wider group, and comparisons between both tumor groups should be performed with extreme caution. According to the National Comprehensive Cancer Network, 17% of diagnosed metastatic colon cancer patients present synchronous peritoneal metastases at diagnosis, of which 2% present metastases restricted to this location. Both locally advanced tumors and the presence of synchronous peritoneal metastases are associated with a worse prognosis with a negative impact on survival [3,4,5].

Due to the improvements in prognosis that are being achieved with some therapeutic strategies in certain clinical contexts, utilizing the correct staging is becoming increasingly important. Some examples of this may be the performance of extended D3 lymphadenectomy in certain colon cancers, especially on the right side, the possibility of administering neoadjuvant chemotherapy in locally advanced cases or even performing surgery with cytoreduction, and hyperthermic intraperitoneal chemotherapy in those cases of tumors with the presence of peritoneal metastases at diagnosis or with characteristics that confer a high risk of local or peritoneal recurrence, such as T4 tumors [6,7,8,9,10,11]. All these treatment options are not yet standard procedures, and therefore, studies are still needed to support their use, especially because they are aggressive treatments with a non-negligible morbi-mortality, which makes the proper selection of patients essential.

However, despite the evolution of therapeutics, the field of diagnostic evaluation in these cases has not undergone the same development. Several recent studies have shown that staging by abdominal CT can be misleading in a percentage of these locally advanced tumors, with a non-negligible risk of errors in preoperative staging, both over- and under-staging, with consequent errors in proper treatment planning [12,13]. The most challenging situations for reaching a correct diagnosis, i.e., some of the scenarios for which the selection of the most appropriate strategy is of utmost importance, can be considered to be the suspicion of a T4 tumor and the presence or absence of affected lymph nodes or peritoneal metastases [14,15,16]. Taking into account the existing difficulties for a correct diagnosis in reference institutions and in clinical studies, the aim of the present study was to analyze the diagnostic reliability of CT for locally advanced colon tumors in a routine clinical practice setting. This analysis could provide valuable information to better select candidate patients for certain aggressive therapeutic strategies in real-life contexts.

## 2. Materials and Methods

Local Clinical Research Ethics Committee (CREC) from Hospital de la Princesa (Madrid) approved this study (04/21-4398).

This study is a secondary analysis of an original study registered in ClinicalTrials.gov, number NCT05300789, in which the oncological outcome was investigated in a selected subgroup of pT4 colon cancer patients [17].

Patient consent was waived due to its retrospective and observational nature.

### 2.1. Design, Patients, and Variables

An observational retrospective multicenter trial was designed. A total of 50 different hospitals were enrolled in the project. This study was sponsored by the Spanish Surgical Society (Asociación Española de Cirujanos), both by the Colorectal and Peritoneal Surgery sections.

All consecutive patients operated on between 2015 and 2017 for colon cancer with curative intent, both elective and emergency operations, with pathological confirmation of pT4 stage adenocarcinoma, were included. Colon cancer was considered as the presence of tumors located in the large bowel at 15 cm above the anal verge. The exclusion criteria were as follows: patients younger than 18 years old, inability to achieve a whole tumor resection or palliative surgery (R2), patients without preoperative CT scan, pathological diagnosis of colon cancer other than adenocarcinoma (such as GIST, leiomyosarcomas, neuroendocrine tumors, or others), and patients with missing information.

Peritoneal metastases were defined a priori in the study protocol as follows: suspicion of any tumoral disease at the peritoneum, either single or multifocal, in the CT scan, and pathologically confirmed peritoneal metastasis as the gold standard for comparison.

Data were recorded by two senior staff members in each participant center. Demographic, preoperative, and operative data as well as pathological analysis based on 8th Edition of TNM classification [18] were recorded.

### 2.2. Evaluation of Radiological Studies

Exact details of the equipment, study protocols, and report were entirely at the discretion of the participating institutions based on best clinical practice [3]. In all but four hospitals, specific teams were appointed to evaluate abdominal radiological complementary tests, including CT scans, for the present study. Every determination acquired during planned CT scans for the staging of colon cancer patients was analyzed and informed within these specific units. Regarding determinations acquired during emergency situations, all of them were reported in this setting, but afterwards, in every institution, all the determinations were routinely re-evaluated by the abdominal imaging team in order to confirm or reinterpret the previously provided information.

### 2.3. Statistical Method

Qualitative variables are presented as numbers with their frequency distribution. Quantitative variables are represented as their mean and standard deviation (SD) or median and interquartile range (IQR) in case of asymmetry. The null hypothesis was rejected when the α or I error was <0.05. In order to assess the CT scan accuracy for the diagnosis of T4 colon malignancies, lymph node status, and peritoneal metastases, the calculated parameters were as follows: sensitivity (S), specificity (Sp), positive and negative predictive values (PPV and NPV, respectively), positive and negative likelihood ratios (PLR and NLR, respectively), and overall accuracy. All parameters are provided with a 95% confidence interval (CI).

The results are reported in accordance with the STARD (Standards for Reporting of Diagnostic Accuracy studies) Statement [19].

All calculations were performed using Stata 13.1 (StataCorp, College Station, TX, USA).

## 3. Results

### 3.1. Patients’ Description and Radiological Findings

**Figure 1:**
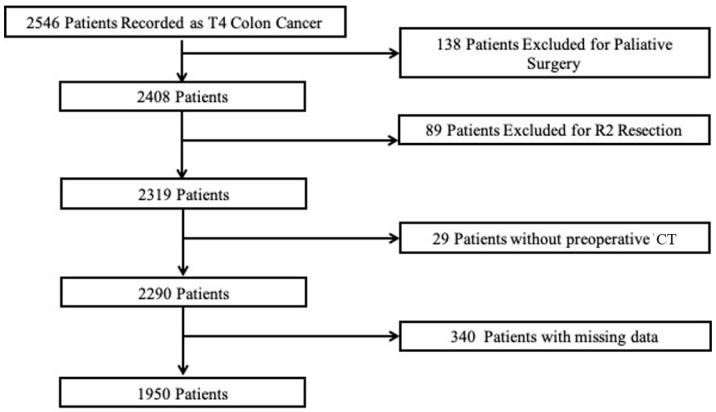
Flowchart detailing the selection of the patients in this study.

A total of 50 different hospitals participated in the study with a total sample record of 2546 patients with pT4 colon cancer. After the inclusion and exclusion criteria were applied, a final population of 1950 patients were analyzed (Figure 1). 

Mean age was 70 years (SD 12 years), and 57% were male patients. Table 1 summarizes demographic, preoperative, and operative variables, and the most relevant postoperative outcomes. The most frequent initial clinical presentations from which colon cancer was diagnosed were obstructive symptoms in 484 cases (29.5%), followed by bleeding in 396 (24.2%), gastrointestinal transit disturbance in 207 (12.6%), and constitutional syndrome in 235 (14.3%). 

All patients underwent preoperative abdominal CT. From the total number of patients, 1110 (56.9%) patients were classified to have T4 tumors, followed by 641 (32.9%) with T3, 114 (5.8%) with T2, and 85 (4.4%) with T0–T1 tumors. Nodal involvement was described in 1061 (54.4%) patients (37.1% N1 and 17.3% N2). Synchronous peritoneal metastases were diagnosed in 78 (4%) cases. Regarding tumor location, 1099 (56.4%) tumors were found in the left colon, with the remainder on the right side. 

### 3.2. Operative Surgical Data and Pathological Assessment

The most common approach was laparotomy, performed in 1196 (61.3%) cases. The most frequent procedure was right hemicolectomy, performed in 802 (41.3%) patients, followed by sigmoidectomy in 581 (29.9%), and left hemicolectomy in 235 (12.1%). Emergency surgery was performed in 465 patients (23.9%) patients of the sample, among them obstruction was the most frequent cause (in 204 patients). A total of 605 (32.4%) patients required extended resection procedures during the intervention.

The histological variables are shown in Table 2. The predominant histological subtype was adenocarcinoma in 1728 tumors (88.6%). Most tumors were low grade (1545; 80.2%) and well (865; 45.5%) or moderately (680; 35.8%) differentiated. The margins were microscopically affected in 221 cases (11.3%). There were 261 (13.4%) cases with a finding of tumor perforation. Within the whole sample, 1487 (76.3%) tumors were T4a, while the remaining tumors were T4b. The mean number of lymph nodes retrieved in the surgical specimens was 21.5 (SD 12.8), with 1652 (84.8%) of the patients having 12 or more nodes evaluated. There were 1233 (63.2%) patients with positive adenopathy for malignancy. After the results of the anatomopathological study, 1396 (71.8%) patients received adjuvant chemotherapy. 

### 3.3. CT Scan Accuracy

The preoperative diagnosis established with abdominal CT on T and N staging (according to lymph node involvement) and the presence of synchronous peritoneal metastases (M1c), in relation to the final pathological diagnosis, is shown in Table 3. Data regarding CT diagnostic accuracy are shown in Table 4. The diagnostic sensitivity and the false negative rate (FNR) were calculated for the “T” staging of these pT4 tumors. The overall sensitivity was 56.9% (95% CI: 52.9–57.7%), while the sensitivity for the diagnosis of tumors categorized preoperatively as locally advanced (T3 and T4) was 89.8% (95% CI 88.5–91.1%).

The values obtained for the diagnostic ability of CT for pathologic lymph nodes (N+) were a sensitivity of 64% (95% CI 61.3–66.6%) and a specificity of 62.1% (95% CI 58.5–65.5%), with an overall accuracy of 63.3%. The positive likelihood ratio for the diagnosis of N+ was 1.7 (95% CI 1.5–1.9), while the negative likelihood ratio was 0.58 (95% CI 0.53–0.64).

For the diagnosis of peritoneal metastases, the sensitivity and specificity were 40% (95% CI 31.7–48.9%) and 98.4% (95% CI 97.7–98.8%), respectively, with an overall accuracy of 94.8%. The positive likelihood ratio for the diagnosis of synchronous peritoneal disease, M1c, was 24.4 (95% CI 17.68–30.5), while the negative likelihood ratio was 0.61 (95% CI 0.53–0.70).

The data about the CT scan accuracy, for stage T4, nodal stage, and peritoneal metastases, are summarized in Table 4. 

## 4. Discussion

In this study, based on a selected cohort of patients with pT4 colon tumors, the most advanced local stage possible, conventional abdominal CT has shown limited diagnostic accuracy, both in the characterization of the T4 stage and in the determination of affected lymph nodes (N+), and also in the diagnosis of synchronous peritoneal metastases.

In an era in which we are moving towards practically individualized medicine and in which patients are intended to be more and more involved in decisions about their treatment, obtaining the best diagnostic accuracy is a fundamental objective, especially because often there is no single standard treatment and the treatment offers and options are constantly increasing.

In the field of advanced colon cancer, certain strategies have flourished, aiming to administer preoperative oncological treatments that were classically administered postoperatively [11]. Although this approach is attractive, it should be noted that it may be associated with a significant risk of over-treatment. Some authors have estimated the risk of over-staging as 1 in every 12 diagnosed patients (95% CI, 9–16) [12]. This should be taken into consideration, not only because of the specific risks of these treatments and their toxicity, but also because of the potential oncological impact on the host, preoperatively altering the dynamics and biology of the tumor. 

Although results are positive when locally advanced tumors in the broadest sense of their characterization (T3 tumors with >5 mm wall involvement and T4 tumors) are considered, the fine differentiation between the different T stages, the diagnostic reliability of the N category, and, above all, the possibility of an accurate diagnosis of the presence of synchronous peritoneal metastases as well as their preoperative characterization, are still unresolved diagnostic challenges.

The findings obtained in the present study have been previously reported by other studies that analyzed this topic, although with clinical designs different from the one chosen in our study [20,21]. 

Fernandez et al. [22] presented a retrospective study in 2019 that determined the diagnostic validity of abdominal CT for T and N staging of colon cancer. They included 150 patients who underwent right hemicolectomy with previous abdominal CT and were divided into two groups (early tumors vs. late tumors). They obtained diagnostic sensitivities of 50% for T1 and T2 tumors and 57% for T3 and T4 tumors. In the case of lymph node staging, the diagnostic parameters (sensitivity, specificity, PPV, and NPV) were 47%, 71%, 59%, and 61%, respectively. In both “N” and “T” staging, underdiagnosis was close to 50%; therefore, they concluded that only using this test for diagnosis could be insufficient. Olsen et al. [23] also retrospectively studied the diagnostic performance of CT, including 4832 colon cancer patients from a Danish national registry. The sensitivity for diagnosing T3-T4 tumors was 73%. The diagnostic parameters for N+ tumors were 57%, 66%, 50%, and 73% for sensitivity, specificity, PPV, and NPV, respectively. According to these results, they recommended caution when making therapeutic decisions based on conventional CT.

A meta-analysis published in 2016 by Nerad et al. [15] studied the sensitivity and specificity of CT for T and N staging; for T staging, they included 13 articles, obtaining 90% and 69%, respectively, while for N staging they included 16 articles, obtaining 71% and 67%, respectively. A differential analysis was carried out according to whether the thickness of the slices used in the CT was greater or less than 5 mm. This analysis provided better results in the second group. In our study, over-staging of tumors ≤ pT3 cannot be estimated as it only includes pT4 tumors. But in pT4 tumors and under real clinical practice conditions, preoperative CT underestimates the T4 category (classifying them as ≤T3) in 43% of cases. As for N, it classifies 36% of pN+ as N0 (false negative—FN). Conversely, 37.9% of pN0 were classified as N+ (false positive—FP). Therefore, both the risk of under-staging and over-staging are relevant for the N category.

Finally, we addressed in our study the diagnosis of synchronous peritoneal metastases. Our results, with a sensitivity of 40%, which means 60% of peritoneal metastases cases remain undiagnosed (FN), and a specificity of 98.4%, which means 1.6% of cases are mistakenly diagnosed to be positive for peritoneal metastases (FP), reflected the existing difficulty in the radiological diagnosis of peritoneal involvement. These figures are quite relevant, as under-estimation of peritoneal metastases might have devastating effects, while over-estimation might not. However, this aspect is the least studied in the literature, with most studies being heterogeneous (including studies of ovarian and other tumors of the upper gastrointestinal tract). Regarding colon cancer, there are few articles published including prospective samples from patients with peritoneal metastases found on abdominal CT who will undergo exploratory laparoscopy, so they calculate the diagnostic parameters with respect to the PCI (peritoneal cancer index). They have reported that CT scan underestimates the peritoneal extension of the disease, with the pelvis being the most underdiagnosed region [24,25,26]. 

In our opinion, it is quite noticeable that the likelihood ratio (LR) has not been used more extensively in the evaluation of CT as a diagnostic tool in this setting. Most common parameters, such as S, Sp, PPV, and NPV values, may not be good measures as they are influenced by the prevalence of the disease or the condition that is being rated in the population. On the contrary, the LR, which represents the ratio of the probability of a particular test result in patients with the disease and the probability of the same result in patients without the disease, is considered a powerful measure of the accuracy of diagnostic tests [25]. Consequently, the interpretation of the LR of a test reflects by how much the probability of presenting a disease increases or decreases depending on the result of a particular test. The closer to one the result of the LR calculations is, the lower is the impact of the diagnostic test employed on the post-test probability of the disease [25]. Based on these considerations, CT scan findings must be still considered with great caution in this context. On the one hand, the high PLR (more than 20) reflects a significant level of correct diagnosis of peritoneal metastases, although on the other hand, a low NLR (0.61) reflects a significant level of underdiagnosis of the disease under consideration. However, this is still more a potential intrinsic problem of the technology resolution and diagnostic capacity of the CT scan itself, more than that of the interpretation of the obtained images. The same problem was highlighted regarding N status based on this interpretation of likelihood ratios.

An accurate preoperative categorization of lymph nodes can be considered a cornerstone for the implementation of certain strategies and to take certain decisions, although it has been demonstrated once again that it is quite difficult to achieve, despite the constant efforts being taken for its improvement. 

To alleviate the problems of diagnostic staging with conventional CT, other techniques have been proposed in recent years for the identification of locally advanced tumors, such as colonography, also known as virtual colonoscopy, PET-CT, and magnetic resonance imaging (MRI). Colonography is indicated in cases with poor endoscopic preparation or high anesthetic risk. According to some studies, this test has a sensitivity of diagnosing more than 80% of locally advanced T tumors. Some authors consider that the distension of the colon applied in this test, and its three-dimensional reconstruction, allow a better evaluation of the deep wall involvement [26]. The other proposed complementary test for the preoperative study of colon cancer is PET-CT. However, this test is not appropriate to determine the in-depth involvement of the intestinal wall since radionuclide uptake seems to be more related to tumor size than to infiltration. It should also be taken into account that its sensitivity and specificity for diagnosing lymph node involvement is close to 40% and 90%, respectively; therefore, its routine preoperative use is not recommended [27]. Finally, the use of MRI in diagnostic studies for the staging of rectal cancer has become routine in recent years. It has also been studied for colon cancer, and it has been found that this test can be more sensitive than conventional CT in discriminating early vs. locally advanced tumors [28,29]. Currently, the evidence is still insufficient to recommend MRI for the therapeutic planning of colon cancer, since the problem of correct lymph node staging is still present [30], and it is a more expensive test, which requires much more time for its performance, and the increase in its demand is difficult to sustain in clinical practice. 

An outstanding issue to study the diagnostic performance of CT for locally advanced colon tumors is the absence of a formal definition of this subgroup of tumors according to the AJCC [31]. Many authors have considered T3 with extramural invasion of >5 mm and T4 as locally advanced tumors, in accordance with what has been used in studies on the effect of neoadjuvant chemotherapy for this tumor group [32,33]. Other studies, on the other hand, consider locally advanced tumors as those classified as T3 and T4, without differentiating the T3 group according to the depth of extramural involvement [21,34]. In the coming years, tools that use artificial intelligence and radiomics will probably be integrated with the current diagnostic means. Today, the use of these instruments is far from its adequate use in clinical practice, but these instruments have the potential to become diagnostic alternatives that may help solve the diagnostic problem in question in the future [35,36].

The study has some limitations such as the fact that it is a retrospective study, there is a lack of information regarding existing protocols for the acquisition and interpretation of the images, and the absence of a common radiological protocol or the lack of information regarding the necessity and accuracy of further diagnostic studies such as MRI or PET-CT in this context, and the aforementioned bias of including only tumors with a histological diagnosis of T4. Taking into account that colon cancer is quite a common condition, it is presumable that all the participating institutions in this study were aware of the best clinical practices and updated guidelines at the time of recording of the data. In addition, although guidelines exist, there is no unanimity in the interpretation of certain images or uniform diagnostic criteria in some circumstances, such as lymph node status or peritoneal metastasis. The present study, developed in a “real-life” setting, is a perfect reflection of the inconsistencies and weaknesses of this important issue, that is, preoperative advanced colon cancer staging. In spite of all this, the study’s strengths are the large number of patients with locally advanced tumors included in this study and that these patients had not received any neoadjuvant treatment before inclusion in this study; therefore, their anatomopathological diagnosis was not altered due to previous therapies. Another novelty of the study is that it not only addresses T and N staging but also addresses the synchronous peritoneal involvement. Finally, we would also like to highlight the use of the likelihood ratio as a parameter of diagnostic utility of CT, as we consider that it is of great diagnostic value and has been scarcely used in the literature up to now.

## 5. Conclusions

The diagnostic accuracy of conventional CT as a preoperative assessment tool for locally advanced colon cancer is still limited for some aspects such as T staging, lymph nodes status, or the presence of synchronous peritoneal metastases. Research to improve preoperative staging should continue, and treatment decisions based on conventional CT should be taken cautiously in view of its risk–benefit analysis.

## Figures and Tables

**Table 1:** Patients´ demographics and preoperative tumor characteristics.

		n	%
Age (Years) *		70.2	(SD 12.3)
Male		1115	57.2
ASA	I	85	4.4
	II	907	46.9
	III	846	43.8
	IV	95	4.9
BMI	<30	1217	77.2
	≥30	359	22.8
Asymptomatic		311	16
Symptomatology	Altered bowel transit	207	12.6
	Obstruction	484	29.5
	Constitutional syndrome	235	14.3
	Bleeding	396	24.2
	Others	317	19.3
Location	Right colon	851	43.6
	Left colon	1099	56.4
cT	T0–T1	85	4.4
	T2	114	5.8
	T3	641	32.9
	T4	1110	56.9
cN	N0	889	45.6
	N1	724	37.1
	N2	337	17.3
cM1		78	4

ASA: American Society of Anesthesiologist; BMI: body mass index. cT: clinical diagnosis for T stage. cN: clinical diagnosis for N stage. cM1: clinical diagnosis for M1 stage. * Data are expressed as mean and standard deviation.

**Table 2:** Operative data, pathological assessment, and adjuvant treatment details.

KERRYPNX		n	%
Type of surgery	Elective surgery	1485	76.2
	Emergency surgery	465	23.9
Surgical procedure	Right hemicolectomy	802	41.3
	Left hemicolectomy	235	12.1
	Sigmoidectomy	572	29.5
	Hartmann’s surgery	136	7
	Others	195	10
Histological type	Adenocarcinoma	1728	88.6
	Signet ring cell/mucinous carcinoma	222	11.4
Differentiation grade	G1	865	45.5
	G2	680	35.8
	G3	357	18.8
Affected margins		221	11.3
Perineural invasion		708	36.9
Vascular invasion		822	42.8
Lymphatic invasion		876	45.8
Tumor perforation		261	13.4
T4a category		1487	76.3
N category	N0	717	36.8
	N1	700	35.9
	N2	533	27.3
M1c		120	6.1
Stage	II	626	32.3
	III	944	48.7
	IV	369	19
Adjuvant chemotherapy		1396	71.8

**Table 3:** CT scan preoperative staging vs. histopathological assessment.

		Pathological Assessment		
		pT4		
CT Scan Diagnosis T category	cT1	85 (4)		
	cT2	114 (6)		
	cT3	641 (33)		
	cT4	1110 (57)		
	TOTAL	1950 (100)		
		**Pathological Assessment**		
		**pN0**	**pN1/N2**	
CT Scan Diagnosis N category	cN0	445	444	889
	cN1/N2	272	789	1061
	TOTAL	717	1233	1950
		**Pathological Assessment**		
		**pM0**	**pM1c**	
CT Scan Diagnosis M1c category	cM0	1800	72	1872
	cM1c	30	48	78
	TOTAL	1830	120	1950

**Table 4:** CT scan accuracy for pT4 tumors, N status, and M1c staging.

T4 Category		
	Value (%)	95% CI
Sensitivity	57	55–59
FNR	43	41–45
**N Category**		
	**Value (%)**	**95% CI**
Accuracy	63	61–65
Sensitivity	64.0	61.3–66.6
Specificity	62.1	58.5–65.5
PPV	74.4	71.7–76.9
NPV	50.1	46.8–53.3
PLR	1.7	1.5–1.9
NLR	0.58	0.53–0.64
**M1c Category**		
	**Value (%)**	**95% CI**
Accuracy	94.8	93.7–95.7
Sensitivity	40	31.7–48.9
Specificity	98.4	97.7–98.8
PPV	61.5	50.4–71.6
NPV	96.2	95.2–96.9
PLR	24.4	16–37
NLR	0.61	0.53–0.70

95% CI: 95% confidence interval; FNR: false negative rate; PPV: positive predictive value; NPV: negative predictive value; PLR: positive likelihood ratio; NLR: negative likelihood ratio.

## Data Availability

All data and materials have been made publicly available through the Mendeley Data repository and can be accessed at Cerdán Santacruz, Carlos (2022), “Metachronous peritoneal carcinomatosis after pT4 colon cancer patients”, Mendeley Data, V2, doi: 10.17632/k28wpghcts.2.

## Supplementary Materials

The following supporting information can be downloaded at: https://www.mdpi.com/article/10.3390/jcm12216764/s1. Spanish collaboration group for the study of metachronous peritoneal metastases of pT4 colon cancer.
