# Supplementary material for: Diagnostic Accuracy of Abdominal CT for Locally Advanced Colon Tumors: Can We Really Entrust Certain Decisions to the Reliability of CT?
Source: J Clin Med. 2023 Oct 26;12(21):6764. doi: 10.3390/jcm12216764 (PMC10648183; doi:10.3390/jcm12216764)
Supplement: Supplementary file 1 [file jcm-12-06764-s001.zip › jcm-2642501-supplementary.pdf]

Title: "Diagnostic accuracy of abdominal CT in locally advanced colon tumours: can we really trust certain decisions to the reliability of CT?".

All the following authors included in the present list have significantly contributed to the present work and have to be identified as authors in Pubmed.

Study collaborators:

Dra. Cristina Rihuete Caro, Hospital Universitario Infanta Elena de Valdemoro (Madrid); Dr. Santos Jiménez de los Galanes Marchán, Hospital Universitario Infanta Elena de Valdemoro (Madrid); Dr. Fernando Fernández López, Hospital Clínico de Santiago (Santiago de Compostela, A Coruña); Dr. Manuel Paz Novo, Hospital Clínico de Santiago (Santiago de Compostela, A Coruña); Dr. Francisco Blanco Antona, Complejo Asistencial Universitario de Salamanca (Salamanca); Dr. José Francisco Noguera Aguilar, Complejo Hospitalario Universitario de A Coruña (A Coruña); Dr. Enrique Moncada Iribarren, Complejo Hospitalario Universitario de Vigo (Vigo); Dr. Pedro Villarejo, Hospital Fundación Jiménez Díaz (Madrid); Dr. Héctor Guadalajara Labajo, Hospital Fundación Jiménez Díaz (Madrid); Dra. Natalia Uribe Quintana, Hospital Arnau de Vilanova (Valencia); Dra. Zutoia Balciscueta Coltell, Hospital Arnau de Vilanova (Valencia); Dr. Juan Beltrán de Heredia, Hospital Clínico Universitario de Valladolid (Valladolid); Dra. Beatriz de Andrés Asenjo, Hospital Clínico Universitario de Valladolid (Valladolid); Dr. Christian Nuño Iglesias, Hospital de Cabueñes (Gijón); Dra. Paola Lora Cumplido, Hospital de Cabueñes (Gijón); Dr. Jesús Bollo Rodríguez, Hospital de la Santa Creu y Sant Pau (Barcelona); Dra. Ane Etxart Lopetegi, Hospital Donostia (San Sebastián); Dr. José Manuel Richart Aznar, Hospital Dr. Peset (Valencia); Dra. Nuria Peris Tomás, Hospital Dr. Peset (Valencia); Dra. Mercedes Estaire Gómez, Hospital General Universitario de Ciudad Real (Ciudad Real); Dra. Esther Garcia Santos, Hospital General Universitario de Ciudad Real (Ciudad Real); Dra. Alicia Calero Amaro, Hospital General Universitario de Elche (Alicante); Dr. Luis Sánchez Guillén, Hospital General Universitario de Elche (Alicante); Dra. Virginia Jiménez Carneros, Hospital General Universitario de Getafe (Madrid); Dra. Ainhoa Valle Rubio, Hospital General Universitario de Getafe (Madrid); Dr. Eduardo de San Pio Carvajal, Hospital Infanta Cristina (Parla, Madrid); Dra. Elena Sagarra Cebolla, Hospital Infanta Cristina (Parla, Madrid); Dra. Araceli Mayol Oltra, Hospital Provincial de Castellón (Castellón); Dr. Enrique Boldó Roda, Hospital Provincial de Castellón (Castellón); Dr. Joaquín Carrasco Campos, Hospital Regional Universitario de Málaga (Málaga); Dr. Alberto Titos García,

Hospital Regional Universitario de Málaga (Málaga); Dra. Estrella Turienzo Santos, Hospital Universitario Central de Asturias (Oviedo); Dra. Isabel Cifrián Canales, Hospital Universitario Central de Asturias (Oviedo); Dra. Arancha Prada, Hospital Universitario de Badajoz (Badajoz); Dra. María Carmona Agúndez, Hospital Universitario de Badajoz (Badajoz); Dr. Manuel Artiles Armas, Hospital Universitario de Gran Canaria Doctor Negrín (Gran Canaria); Dra. Beatriz Arencibia Pérez, Hospital Universitario de Gran Canaria Doctor Negrín (Gran Canaria); Dra. Lara Blanco Terés, Hospital Universitario de la Princesa (Madrid); Dr. Álvaro Gancedo Quintana, Hospital Universitario de la Princesa (Madrid); Dr. José Antonio Rueda Orgaz, Hospital Universitario Fundación Alcorcón (Madrid); Dr. Federico Ochando Cerdán, Hospital Universitario Fundación Alcorcón (Madrid); Dr. Manuel Díez Alonso, Hospital Universitario Principe de Asturias (Madrid); Dra. Remedios Gómez Sanz, Hospital Universitario Principe de Asturias (Madrid); Dr. Juan Ocaña Jiménez, Hospital Universitario Ramón y Cajal (Madrid); Dr. Julio Galindo Álvarez, Hospital Universitario Ramón y Cajal (Madrid); Dra María del Mar de Luna Díaz, Hospital de Pozoblanco (Córdoba); Dr. Enrique Asensio Díaz, Hospital Universitario Rio Hortega (Valladolid); Dr. Fernando Labarga Rodríguez, Hospital Universitario Rio Hortega (Valladolid); Dra. Marta Allué, Hospital Universitario San Jorge (Huesca); Dra. Alejandra Utrilla Fornals, Hospital Universitario San Jorge (Huesca); Dra. Inmaculada Segura Jiménez, Hospital Universitario Virgen de las Nieves (Granada); Dra. Raquel Conde Muíño, Hospital Universitario Virgen de las Nieves (Granada); Dra. Tamara Fernández Miguel, Hospital Universitario Galdakao Usansolo (Bilbao); Dra. Irune Vicente Rodríguez, Hospital Universitario Galdakao Usansolo (Bilbao); Dra. María Conde Rodríguez, Hospital Universitario Lucus Augusti (Lugo); Dra. Laura Ramírez Ruiz, Hospital Universitario Lucus Augusti (Lugo); Dra. Beatriz Moreno Flores, Hospital General Universitario de Albacete (Albacete); Dra. Cristina Camacho Dorado, Hospital General Universitario de Albacete (Albacete); Dr. Juan Torres Melero, Complejo Hospitalario Universitario Torrecárdenas (Almería); Dr. Miguel Lorenzo Liñán, Complejo Hospitalario Universitario Torrecárdenas (Almería); Dra. María Labalde Martínez, Hospital Universitario 12 de Octubre (Madrid); Dr. Francisco Javier García Borda, Hospital Universitario 12 de Octubre (Madrid); Dra. Vanessa Concepción Martín, Hospital Universitario Nuestra Señora de la Candelaria (Tenerife); Dra. Carmen Díaz López , Hospital Universitario Nuestra Señora de la Candelaria (Tenerife); Dr. Pedro A. Parra Baños, Hospital Universitario Reina Sofía (Murcia); Dr. Emilio Peña Ros, Hospital

Universitario Reina Sofía (Murcia); Dra. Mireia Amillo Zaragüeta, Hospital General de Granollers (Barcelona); Dr. Orlando Aurazo, Hospital General de Granollers (Barcelona); Dr. David Alías Jiménez, Hospital Universitario Rey Juan Carlos (Madrid); Dra. Rocío Franco Herrera, Hospital Universitario Rey Juan Carlos (Madrid); Dr. Antonio Ramos Bonilla, Hospital Severo Ochoa de Leganés (Madrid); Dra. Marta Pérez González, Hospital Severo Ochoa de Leganés (Madrid); Dra. Asunción Pérez Sánchez, Hospital Universitario Virgen Macarena (Sevilla); Dr. Javier Valdés Hernández, Hospital Universitario Virgen Macarena (Sevilla); Dra. Laura González Sánchez, Complejo Hospitalario Insular-Materno Infantil (Las Palmas); Dr. Eudaldo López-Tomasetti Fernández, Complejo Hospitalario Insular-Materno Infantil (Las Palmas); Dr. Carlos Fernández Mancilla, Hospital San Juan (Alicante); Dra. Cristina González Prado, Hospital Universitario de Burgos (Burgos); Dr. Guillermo Cabriada García, Hospital Universitario de Burgos (Burgos).
